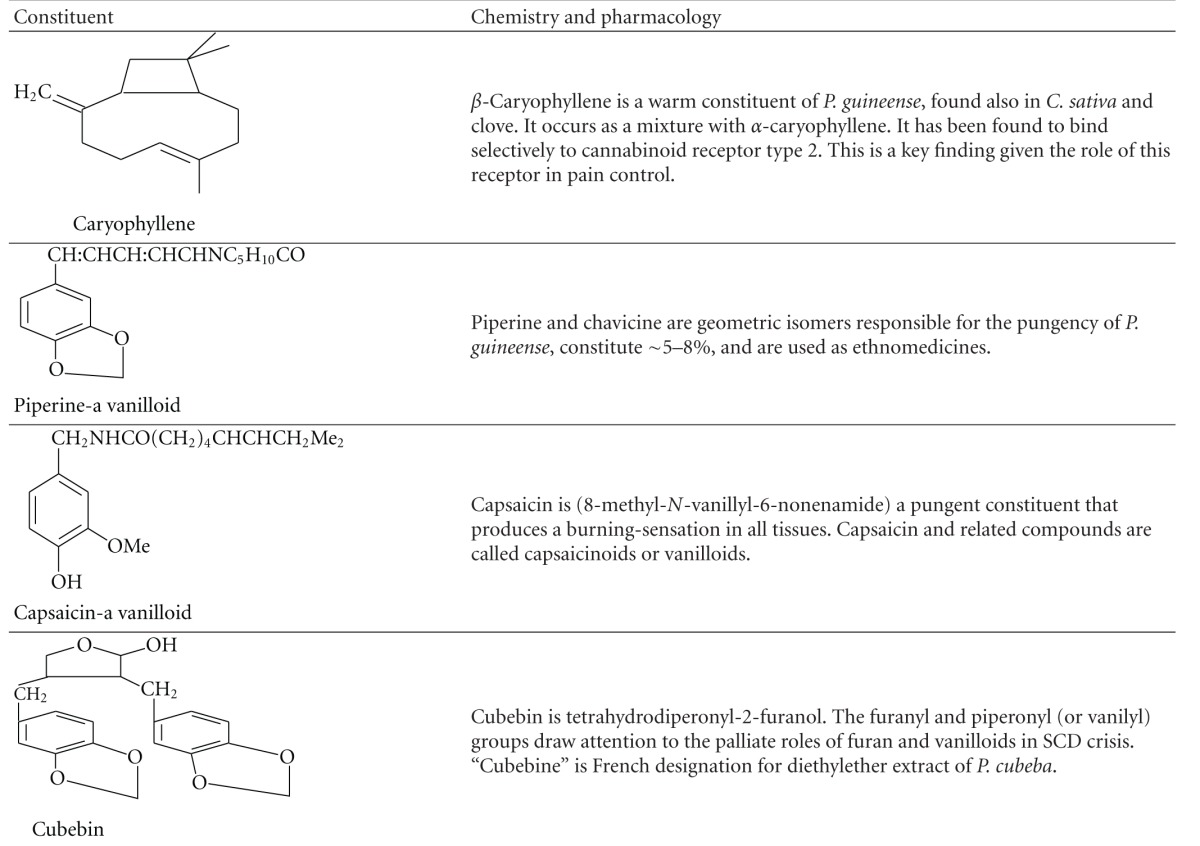# Traditional Herbal Management of Sickle Cell Anemia: Lessons from Nigeria

**DOI:** 10.1155/2012/607436

**Published:** 2012-11-08

**Authors:** Sunday J. Ameh, Florence D. Tarfa, Benjamin U. Ebeshi

**Affiliations:** ^1^Department of Medicinal Chemistry and Quality Control, National Institute for Pharmaceutical Research and Development (NIPRD), PMB 21, Garki, Idu Industrial Area, Abuja, Nigeria; ^2^Department of Pharmaceutics & Medicinal Chemistry, Niger Delta University, Wilberforce Island, Amassoma, Nigeria

## Abstract

*Background*. Patients in West Africa where sickle cell anemia (SCA) is endemic have for ages been treated with natural products, especially herbs, as, is still the case in rural communities. *Objective*. In this paper we look closely at some of these herbs to see if there are any lessons to be learnt or clues to be found for optimizing the treatments based on them, as had been done in the case of NIPRISAN, which was developed from herbs in Nigeria based on Yoruba Medicine. *Methods*. Select publications on SCA, its molecular biology and pathology, and actual and experimental cases of herbal treatment were perused in search of molecular clues that can be linked to chemical constituents of the herbs involved. *Results*. The study revealed that during the last 2-3 decades, much progress was made in several aspects of SCA pharmacology, especially the approval of hydroxyurea. As for SCA herbalism, this paper revealed that antisickling herbs abound in West Africa and that the most promising may yet be found. Three new antisickling herbs (*Entandrophragma utile*, *Chenopodium ambrosioides*, and *Petiveria alliacea*) were reported in May 2011. At NIPRD, where NIPRISAN was developed, three other recipes are currently awaiting development. *Conclusion*. The study raised the hope that the search in the Tropics for more effective herbal recipes for managing sickle cell anaemia will be more fruitful with time and effort.

## 1. Introduction 

### 1.1. Health and Disease as Conceived among Communities in Nigeria

**Figure 1:**
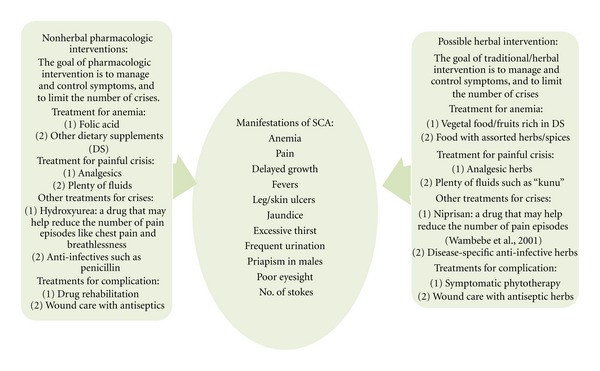
Manifestations of SCA and strategies for management including herbal treatment. The two major pathologies of SCA are hemolytic anemia and vasoocclusion with pain especially in the limbs. Acute chest syndrome, which can result from infections, is the leading cause of death. Neurologic complications such as stroke and hemorrhage can occur. Aplastic crisis is most often the result of infection with Parvovirus B19, which results from temporary cessation of RBC production. Genitourinary-hematuria, renal failure, and priapism may occur. Cholelithiasis due to severe hemolysis can develop into acute cholecystitis due to the formation of pigmented gallstones [6–13].

Health and disease concepts in African Traditional Medicine are far more advanced than many biomedical scientists would imagine. For instance, long before Ronald Ross revealed mosquito as the vector of malaria and Charles Laveran plasmodium as the parasite [1], communities in tropical African had associated mosquitoes with high fever. Among the Idoma of Benue State, Nigeria, it was known since antiquity that “idapo” (malarial fever) is caused by “imu” (mosquito) and that “ofe-egbe” (dysentery) is caused by bad water or eki-iju (egg of green house flies: a variety of *Musca domestica* associated with poor sanitation). On the other hand, disorders underlain by more remote causes are attributed to evil spirits and practices frowned upon or forbidden by tradition. Such practices include marriage between close relatives. In Idomaland, marriage even between second cousins is expressly forbidden—it is considered an abomination and a cause of abnormalities or incurable disorders. We are not aware of any specific name for sickle cell disorder in Idoma, but we know that the condition is common and is classed among diseases believed to be caused by evil spirits or misconduct. Ibrahim Muazzam, NIPRD's ethnobotanist and an associate of Etkin [2], informed us that among the Hausa-Fulani of northern Nigeria, where sickle cell anemia is called “sankara-miji,” the disorder is perceived to be “paranormal” and incurable. Among the Yoruba and Igbo of southern Nigeria, “Abiku” [3] and “ogbanje” [4] or “iyi-uwa” [5] are umbrella terms that include sickle cell anemia and are believed to be “paranormal”. The foregoing suggests to us that traditional communities in Nigeria are not only aware of the syndrome called “sickle cell anemia” but also well aware of its chronicity, endemicity, and “paranormality.” The general manifestations of SCA and strategies for management including herbal treatment are indicated in Figure 1.

### 1.2. Traditional Herbal Approaches to Sickle Cell Anemia in Nigeria

As described elsewhere [14, 16, 17], among the Efik and Ibibio, Hausa, Igbo, Idoma, and Yoruba: clove (*Eugenia caryophyllata* or “kanunfari” in Hausa; *Piper guineense* (“eche” in Idoma or “akwa-ose” in Igbo); grains of paradise (*Aframomum melegueta *or “otuta” in Idoma); *Sorghum bicolor *(the leaf stalk yields an extract that looks like blood); *Pterocarpus osun *(common in the Yoruba state of Osun) are used in various health conditions, including sickle cell anemia. As stated earlier [14] *E. caryophyllata*, *P. guineense*, *P. osun*, and *S. bicolor *are the herbal components of the Yoruba recipe upon which the antisickling drug Niprisan is based. Prior to the era of Niprisan these herbs were either extracted with “ogoro” (ethanolic distillate of palm wine) or with an aqueous solution trona (sodium sesquicarbonate—a mineral used in Nigeria as tenderizer). Niprisan has passed phases IIA and IIB, and is widely used in Nigeria, and is known or popular in India and the USA. In 2010, Swift [18] of COSMID Corporation, USA, stated the following:

 A dried extract of four plants has been used to treat patients with SCD in Nigeria for many years (NIPRISAN). It has been through multiple clinical trials in Nigeria and has been formally approved for use in that country since 2006 for the treatment of SCD. The US FDA has determined there is sufficient safety and efficacy data for NIPRISAN to start a Phase III clinical trial. The US FDA Botanical Review Team (BRT) suggested a simpler formulation of NIPRISAN, development of a chemical fingerprint for the formulation using LC/MS and elucidation of some of the anti-sickling compounds in the formulation would improve standardization and increase the probability of obtaining FDA marketing approval.

**Figure 2:**
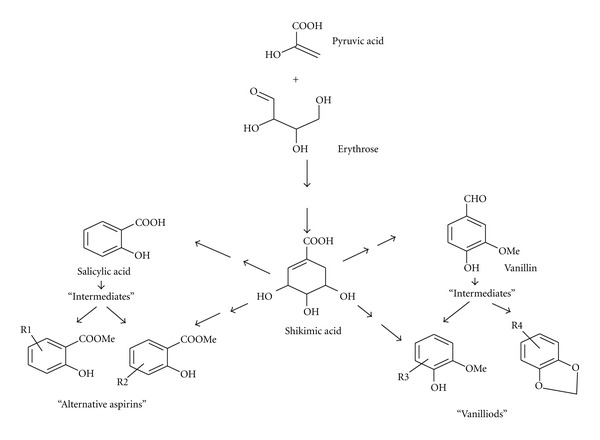
Biosynthesis and relationship of shikimic acid to “alternative aspirins” and “vanilloids”. The shikimic acid pathway is a key biosynthetic pathway for several phytochemicals known for their medicinal attributes. The Figure illustrates the biosynthesis of shikimic acid from pyruvic acid and erythrose and the relationship between the acid and its byproducts and intermediates, some of which possess aspirin-like effects, like analgesia and desickling of sickled RBCs. Such byproducts/intermediates include salicyclic acid derivatives, vanillin, piperine, capsaicin, and cubebin. Piperine, capsaicin, and cubebin as byproducts of shikimic acid are the likely antisickling agents Niprisan [14]. It is of note that Ouattara [15] had attributed the antisickling properties *Fagara zanthoxyloides* to divanilloylquinic acids.

To the best of our knowledge phase III trial of Niprisan is yet to be reported. We did however suggest in 2011 that phytocannabinoids and vanilloids in *E. caryophyllata* and *P. guineense *may account for some of the useful effects of Niprisan in sickle cell crisis [14]. Some of these compounds, including shikimic acid derivatives (vanilloids) and cannabinoids are indicated in Figure 2 and Table 3, respectively.

### 1.3. An Overview of Vanilloids and Cannabinoids—Agents in Pain Control

The probable roles of vanilloids and cannabinoids receptors in the control of pain, the key issue in sickle cell crisis, are described in a latter section of this paper. In the present we briefly mention these groups of phytochemicals and their synthetic analogues as components of *E. caryophyllata* and *P. guineense, *and that they may account for some of the useful effects of Niprisan in sickle cell crisis [14].

#### 1.3.1. Vanilloids

The vanilloids, namely: vanillin, eugenol, zingerone, capsaicin, and piperine (isomer of capsaicin), are molecules with distinctive flavours, yet are quite similar in their molecular structures. All contain a benzene ring. Subtle changes in the sizes or positions of groups of atoms attached to the ring dramatically change their organoleptic and other physicochemical characteristics. Eugenol, capsaicin, and piperine are present in *E. caryophyllata* and *P. guineense*. Eugenol has a short hydrocarbon chain attached to the ring, which makes it much less water soluble than vanillin. Although it is practically insoluble in water, it freely mixes with fats and oils. Its fat solubility allows it to penetrate tissues and bind more tightly to lipid rich membrane bound vanilloid receptors. The tail gives eugenol a stronger odour than vanillin. Eugenol has a numbing analgesic effect; because it has some antiseptic effects it is used in the formulation of some brands of toothpaste. It is supposed that the hydrocarbon tail in combination with the polar OH group on the ring allows eugenol to interact with vanilloid receptors in order to produce analgesia and other physiochemical effects. Capsaicin and piperine are of a lower molecular weight than eugenol, and their side chains contain a polar amide group, which makes them less volatile and almost odourless but very “hot”—a persistent burning sensation even at concentrations lower than 10 ppm. The intense flavour results from the molecules' long hydrocarbon tails. The chain allows them to bind very strongly with their membranous lipoprotein receptors. The fatty tail also allows the molecules to slip through lipid-rich cell membranes, making the burning sensation more pervasive and persistent. Both the burning and analgesic effects of capsaicin or piperine owe to the way the molecules interact their lipoprotein receptors. Paradoxically, the ability of these compounds to cause pain (i.e., the burning sensation) makes them useful in alleviating pain. Exposure to them lowers sensitivity to pain, and it is applied as a counterirritant in the treatment of arthritis and other chronically painful conditions. People who use lots of pepper (as the Idoma and Yoruba do) build up a tolerance to it. According to Fred Senese [19]—“a small jolt of capsaicin excites the nervous system into producing endorphins, which promote a pleasant sense of well-being. The endorphin lift makes spicy foods mildly addictive (and for some, an obsession).” 

#### 1.3.2. Cannabinoids

Cannabinoids are a group of terpenophenolic compounds present in *Cannabis sativa*' and occur naturally in the nervous and immune systems of animals. The broader definition of cannabinoids refers to a group of substances that are structurally related to tetrahydrocannabinol (THC) or that bind to cannabinoid receptors. The chemical definition encompasses a variety of distinct chemical classes: the classical cannabinoids structurally related to THC; the nonclassical cannabinoids—the aminoalkylindoles, eicosanoids—related to the endocannabinoids 1, quinolines, and arylsulphonamides; additional compounds that do not fall into these standard classes but bind to cannabinoid receptors (CB_1_ and CB_2_). An example of such is *β*-Caryophyllene, which binds selectively to CB_2_. Currently, there are three general types of cannabinoids: the “phytocannabinoids” that occur uniquely in the cannabis plant or the caryophyllenes that occur in clove; the “endogenous cannabinoids” that are produced in the in humans and other animals; the “synthetic cannabinoids” that are similar compounds produced by in the laboratory by chemical manipulations [20].

### 1.4. Aim of the Paper

Eleven years into the 21st century, the only cure for SCA is bone marrow transplant that requires a rigorously compatible family member as donor. There is at best only an 85% disease-free survival rate, with a 7% transplant-related mortality rate and a 9% graft failure rate [21]. The procedure is expensive and precarious, and suitable donors are hard to come by. Moreover, patients needing the treatment the most are the least likely to benefit from it due to higher risks. These barriers mean that pharmacologic approaches (and that include herbal palliation) will remain the primary strategy for managing SCA. This paper is necessitated by the mistaken notion that herbal remedies are coming too late in the day to feature significantly in SCA management. We are inclined to reason otherwise and to hold the view that a thorough familiarity with SCA and the ever increasing volume of data on phytochemicals may provide valuable leads, lessons, and clues. In this regard, we here wish to draw attention to the following pertinent comment [22]:

 Doctors in Nigeria use fagara (*F. zanthoxyloides*) to reduce the painful crisis of the genetic disease, sickle cell anemia. This herb has a variety of unusual properties that reduce platelet and blood cell sticking. After reading the reports from Nigeria many years ago, I decided to try fagara's relative prickly ash bark for the same indication. I made a simple tincture of 50% prickly ash bark and 50% ginkgo leaf, and gave it to a young African-American girl in the first grade who constantly missed school and needed to be hospitalized 3-4 times per year due to the painful sickle cell crisis. I gave her about 25 drops three times a day. She immediately stopped having serious problems, her thinking was no longer fuzzy, the frequency of her attacks went down to about one per year, and the severity of the attacks decreased appreciably. This success has continued through the years, as long as she takes her medicine. I saw her last year, and she has blossomed into a beautiful junior high school student, the sickle cell disease now only a bit-player in the background of her life. Another of my patients had lived with the disease his entire life, with almost constant pain, and bimonthly crisis. I gave him 35 drops three times per day, and he immediately improved in the same way as the young girl. This improvement in both frequency of attacks and level of pain has persisted in three of my long-term patients over many years. The wholesale cost of this medicine is less than $20 per month at full dosage. My biggest fear is that this knowledge will be co-opted by a pharmaceutical company, and made available to the many suffering children only at an exorbitant cost.

Elsewhere, we had reasoned on the need for support for clinical trials of promising traditional remedies, and for national drug regulatory agencies in developing countries to show more interest in herbal clinical trials [17]. We attempt in this paper to show how this line of thought should follow from what we have learnt of herbal management of SCA in Nigeria.

## 2. An Overview of SCA

### 2.1. Epidemiology of SCA and Some Historical Landmarks

Epidemiology of SCA commenced in the USA in 1910 with the discovery of the disorder in a patient hospitalized in 1904, suffering from anemia [41]. The study progressed through the era involving Pauling and others [42] and Ingram [43, 44]; and is today a success because the necessary preventive measures for SCA are now well known [45]. Also fairly documented is the use in Africa of herbal palliatives for SCA [24, 25, 46]. In this paper we take a closer look at some of these herbs and hypothesize on the likely biochemical bases for their use and how such insight may facilitate their optimization. Ages before European colonization of West Africa, the people had identified a chronic condition variously called “Abiku,” “Ogbanje,” “Sankara-jimi” in Nigeria. Thus, several therapies—herbal and otherwise—waxed and waned, but most of the herbals survived. Shortly after SCA was defined in the US, studies at Ibadan University confirmed the syndrome in Nigeria. During the 1970s studies at Ibadan and Ife described the first series of herbal remedies for SCA. In the 1990s biomedical scientists from Ibadan, Ife, and Zaria developed Niprisan which was launched in 2006. In 2001, before the franchise to produce Niprisan was licensed to a US drug firm, NIPRD had 3 other promising recipes. It should be mentioned that the disease was first named “sickle-cell anemia” in 1922 [47]. But some elements of the disorder had been recognized in an 1846 paper in Southern Journal of Medical Pharmacology which described the absence of a spleen in the autopsy of a runaway slave [48, 49].

### 2.2. Prevalence, Manifestations, and Management Strategies

#### 2.2.1. Global Prevalence of SCA

Aside from Africa and countries bordering the Mediterranean (e.g., Italy, Greece, Spain, and Turkey) that have high incidences of SCA, significant prevalence has been reported especially in Saudi Arabia, Yemen, India, Pakistan, Bangladesh, and China [6, 23, 26, 50, 51]. The occurrence of SCA in the Americas and in Northwest Europe owes of course to the Triangular Slave Trade [23].  Table 1 shows the general picture of sickle cell disorders (SCDs) worldwide. 

#### 2.2.2. Manifestations of SCA and Strategies for Management

Key manifestations of SCA are indicted in Figure 2 with comments on symptoms treated with pharmacologic agents and nonpharmacologic strategies. Pharmacologic agencies, of course, include herbal preparations such as Niprisan or Ciklavit.

## 3. Herbal Materials Used in Managing SCA

### 3.1. Examples of Plants Used in Managing SCA

A summary of the gross effects and the proposed general actions of some of the herbs used in SCA treatment is presented in Table 2. 

## 4. Biochemical Bases for Herbal Management of SCA 

### 4.1. Structure of Hemoglobin in Relation to Antisickling Agents

Hemoglobins exist in two quaternary states—the deoxygenated conformation called Tense or T-state and the oxygenated conformation called Relaxed or R-state. Sickling occurs only in T-state haemoglobin S (HbS) due to its polymerizing tendency. Thus, a key approach to the crisis of sickling lies in finding a means of inhibiting this tendency of T-state HbS or of causing it to revert to the R state. Safo and coworkers [52] had shown that both HbA and HbS possess allosteric sites with which suitable chemical ligands can interact to shift the equilibrium in favor of the R state and have identified several such entities, called allosteric regulators. These regulators in the case of HbS act as antisickling agents—which can be defined as entities that can inhibit or reverse the sequence of pathological processes leading to sickling. Compounds known to possess this type of effect include (i) “alternative aspirins” such as acetyl-3,5-dibromosalicylic acid [53], (ii) furfural derivatives [52], and (iii) a variety of compounds called capsaicinoids or vanilloids that possess a vanilyl functional group, or its approximation as in vanillin or related compounds [54]. These vanilloids include some substituted benzaldehydes [55] and several shikimic acid byproducts. The structures of some of these antisickling entities including the “alternative aspirins” and “vanilloids” are shown Figure 2. 

### 4.2. Physical Pain and Biochemical Bases for Its Amelioration

Physical pain is an unpleasant sensation associated with actual or potential tissue damage and is essential to an organism's defense and coordination. But, since pain tends to persist beyond its immediate purpose, organisms are equipped with endogenous systems for controlling pain. Such systems are orchestrated by a complex interplay of ion channels and receptors [56].

#### 4.2.1. Ion Channels

Ion channels are pore-forming proteins that act to establish and control voltage gradient across the plasma membrane of cells by allowing the flow of ions across their electrochemical gradient [56]. A special group called transient receptor potential (or TRP) channels has 28 members that differ in the way they are activated. Some are constitutively open, while others are gated by voltage, ligands, pH, redox state, osmolarity, heat, or mechanical stretch [15]. 

#### 4.2.2. Vanilloid or Capsaicin Receptor (TRPV1) as an Ion Channel

The so-called transient receptor potential vanilloid (TRPV) group of channels has 6 subfamilies, designated-TRPV1 to TRPV6 [15, 57–59]. Caterina et al. [60] indicate that TRPVs are so sensitive to temperature that they are regarded as molecular thermometers. TRPV1 is activated at 43°C and by acidic pH, allicin in garlic, vanilloids (e.g., piperine and capsaicin) and by endocannabinoids (e.g., anandamide and N-arachidonoyl-dopamine). To illustrate how the vanilloids act, it has been shown that capsaicin selectively binds to TRPV1 on the membrane of pain or heat sensing neurons [60]. As a heat activated calcium channel, TRPV1 normally opens at 37–45°C. However, when capsaicin binds to TRPV1, it causes the channel to open below 37°C (body temperature), which is why capsaicin is linked to the sensation of heat. Prolonged activation of these neurons by capsaicin leads to a depletion of presynaptic substance P—a neurotransmitter for pain and heat. Neurons lacking TRPV1 are unaffected by capsaicin [59, 60]. 

#### 4.2.3. Cannabinoid Receptors in Pain Control

The cannabinoid receptors are a class of cell membrane proteins that are activated by lipids called cannabinoids [61]. Some cannabinoids are endogenous, while others (e.g., the psychoactive constituents of *Cannabis sativa*) are exogenous. At least two receptor subtypes—CB_1_ (expressed mainly in the CNS, lungs, liver, and kidneys) and CB_2_ (expressed in the immune system, hematopoietic cells, and peripheral nerve terminals where they function in pain control)—are known. All these tissues are involved in SCA crisis. 

#### 4.2.4. *β*-Caryophyllene: a Component of *P. Guineense* and Clove Is a Cannabinoid

*β*-Caryophyllene, a constituent of *Cannabis sativa* and of *E. caryophyllata* and *P. guineense*: components of Niprisan, has been found to bind selectively to CB_2_, and to exert significant cannabimimetic effects in mice [62]. This implies that caryophyllene can relief pain in humans and be of benefit to SCD patients. Hence it is possible that Indian hemp, which is richer in caryophyllene than Niprisan, may someday be developed for SCA medication.

## 5. Conclusion

This paper revealed that antisickling herbs are common in West Africa and that more are still being discovered. At NIPRD, where some aspects of Niprisan are still being researched, there are currently three other recipes earmarked for development. We figure that with time more effective antisickling herbs will be found and developed if proper strategies are instituted.

## Figures and Tables

**Table 1:** Significant cases of SCDs including thalassemias by continent/region.

Continent/region	Major disorder	Remark/reference
Africa	(1) SCA (HbSS) (2) HbSC (3) *α*-Thalassemia HbC has lysine rather glutamine in 6th position as in *β*-globin of HbA	One in 12 Blacks worldwide carries the SCA trait. About 1 in 400 has SCA. About 75% of global SCAs are in Africa. About 150,000 SCA cases are born yearly in Nigeria. The carrier frequency ranges between 10% and 40% across equatorial Africa, decreasing to 1-2% in north Africa and <1% in South Africa [6].

Islands and countries in Mediterranean area and the Middle East	(1) HbS*β* ^0^ or *β* (2) α-Thalassemia (3) *β*-Thalassemia (4) HbC*β* ^0^ or *β* ^+^ (5) SCA	These islands and countries including Turkey have significant cases of SCDs and thalassemias. Saudi Arabia has a yearly rate of ~3,000 newborns. Qatif City has the highest rate [7, 8].

America—USA	(1) SCA (2) *β*-Thalassemia (3) HbC*β* ^0^ or *β* ^+^ (4) Other SCDs	About 72,000 persons in the US have SCA, mostly African-Americans at the rate of 1 in 500 newborns as against 1 in 1, 200 for Hispanic-American births [23, 24]. In 2004, 83,149 cases of hospitalization were attributed to SCD in the US at a cost of ~$488 million [9].

Asia	(1) SCA (2) *α*-thalassemia (3) *β*-thalassemia (4) HbC*β* ^0^ or *β* ^+^ (5) HbE*β* ^0^ or *β* ^+^ HbC has lysine rather glutamine in 26th position as in *β*-globin of HbA	SCA is significantly prevalent in Bangladesh, China, and other Asian countries. In India the prevalence ranges from 9.4 to 22.2%. Hemoglobin E/thalassemia is common in Cambodia, Thailand, and India. The Maldives has the highest incidence of thalassemias in the world with a carrier rate of 18%. The corresponding figures for Bangladesh, China, India, Malaysia, and Pakistan range 3–8% of the populations [7, 10].

Europe	(1) *β*-thalassemia (2) *α*-thalassemia (3) HbC*β* ^0^ or *β* ^+^ (4) HbE*β* ^0^ or *β* ^+^ (5) SCA	Aside from well-known cases in Italy, Greece, Portugal and Spain, significant prevalence of SCDs and the thalassemias occur in others. In UK more than 200 babies are born annually with SCD. The highest prevalence of 1 in 2,415 is in France due to immigration from more endemic zones [10].

New SCDs/1000 in select areas: Nigeria: ≥19 Ghana: 10–18.9 S. Arabia: 5–9.9 Europe: ≤0.1	Types of SCD seen: (1) *β*-thalassemia (2) *α*-thalassemia (3) HbC*β* ^0^ or *β* ^+^ (4) HbE*β* ^0^ or *β* ^+^ (5) SCA	New SCDs/1,000 in selected areas [7, 12, 25, 26]: Mexico: 0.1–0.19 Central America: 1–18.9 South America: 0.1-4.0 Southeast Asia: 0.2–18.9 Oceania: ≤0.1

*α*-Thalassemia results froms decreased production of *α*-globin leading to an excess of *β*-globin in affected adults or an excess of *γ*-globin in affected newborns. The excess *β*-globin form unstable tetramers called Hemoglobin H (or HbH) consisting of 4 *β*-globin chains that exhibit abnormal oxygen dissociation curves. *β*-Thalassemias are either of the *β* ^0^ type (thalassemia major) or of the *β* ^+^ type (thalassemia intermedia). In the *β* ^0 ^type-thalassemias there is no production of *β*-globin; hence it is the severer form of *β*-thalassemia. In the *β* ^+^ type thalassemia some *β*-globin is produced, making it in the milder form. In either case, however, there is a relative excess of *α* chains, but these do not form tetramers; instead, they bind to RBC membranes, producing membrane damage, and at high concentrations they form toxic aggregates that lead to anemia. As indicated in the table thalassemias can coexist with SCDs.

**Table 2:** Herbal materials used in managing SCA and its probable modes of action.

Herb/reference	Probable general effect/mode of action/phytochemical constituents
*Fagara zanthoxyloides* (root) [27, 28]	Three isomeric divanilloylquinic acids (burkinabin A, burkinabin B, and burkinabin C) were identified as the likely antisickling agents. But some workers have proposed coumarins, vanillic acid, parahydroxybenzoic acid, and paraflurobenzoic acid.

*Carica papaya*—(unripe fruit or leaf) [29–31]	Antisickling effects of 87% inhibitory and 74% reversal activities were obtained from the 5-day fermentation of unripe fruit of *C. papaya* at 2.5 mg per mL of water. Methanol extract had 64% inhibitory and 55% reversal activities while the chloroform extract was inactive. Phenylalanine, tyrosine, and glycine were thought to be responsible.

*Garlic* (bulb) [32]	The basis is unknown, but allicin in garlic, is a potent stimulus of TRPV1 as mentioned in Section 4 (Biochemical Bases for Herbal Management of SCA). Moreover, garlic is used in many infective conditions especially respiratory infections in SCA.

*Hymenocardia acidai* (leaf) [33]	Mpiana et al. [33] related the anti-SCA activities of *H. acida* to anthocyanins.

*Cajanus cajan* (seed) [34, 35]	Phenylalanine is thought to be the most active principle in *Cajanus cajan* seed—a component of Ciklavit antisickling phytomedicine, developed in Nigeria by two professors, Ekeke and Shode [34].

*Khaya senegalensis* (stem bark/leaf) [36]	Fall et al. [36] attributed the anti-SCA effects of *K. senegalensis* to limonoids.

The herbs Niprisan: (1) *S. bicolor* (2) *P. osun* (3) *Clove* (4) *P. guineense* [37–40]	The bases for the actions of *Sorghum bicolor* and *Pterocarpus osun* are unknown, but they are rich in brightly coloured red/orange flavonoids. They probable act as hematonics especially if they contain folic acid or its analogues. Given their blood red colour, the “Doctrine of Signatures” as mentioned elsewhere [16, 17] may have influenced their inclusion by Yoruba sages of old. It had been supposed that the principles in Niprisan that mitigate, palliate, or reduce the frequency of SCA crisis [18] probably reside mainly in clove and *P. guineense* [14].

Clove is Eugenia caryophyllata, which, like *P. guineense*, contains principles that impact SCA crisis. Notably, the isomeric divanilloylquinic acids of *Fagara zanthoxyloides* contain the vanillyl group as do the vanilloids of clove and *P. guineense*. A discussion of these principles is presented in Section 4.

**Table 3:** Some bioactive agents of *P. guineense and E. caryophyllata*—components of Niprisan.